# The prevalence and evidence-based management of needle fear in adults with chronic disease: A scoping review

**DOI:** 10.1371/journal.pone.0253048

**Published:** 2021-06-10

**Authors:** Emily Duncanson, Richard K. Le Leu, Lisa Shanahan, Luke Macauley, Paul N. Bennett, Rick Weichula, Stephen McDonald, Anne L. J. Burke, Kathryn L. Collins, Anna Chur-Hansen, Shilpanjali Jesudason

**Affiliations:** 1 Central and Northern Adelaide Renal and Transplantation Service, Royal Adelaide Hospital, Adelaide, South Australia, Australia; 2 School of Psychology, Faculty of Health and Medical Sciences, University of Adelaide, South Australia, Australia; 3 Department of Medicine, University of Adelaide, Adelaide, South Australia, Australia; 4 Paramount Health Service, Adelaide, South Australia, Australia; 5 Patient Partner for Central and Northern Adelaide Renal and Transplantation Service Clinical Research Group, Royal Adelaide Hospital, Adelaide, South Australia, Australia; 6 Clinical and Health Services, University of South Australia, Adelaide, Australia; 7 Centre for Evidence-based Practices, South Australia, Faculty of Health and Medical Sciences, University of Adelaide, South Australia, Australia; 8 Psychology Department, Royal Adelaide Hospital, Adelaide, South Australia, Australia; University of Maryland School of Medicine, UNITED STATES

## Abstract

**Background:**

Little is known about the prevalence and best management of needle fear in adults with chronic disease, who may experience frequent and long-term exposure to needles for lifesaving therapies such as renal dialysis and cancer treatment. Identifying interventions that assist in management of needle fear and associated distress is essential to support these patients with repeated needle and cannula exposure.

**Method:**

We followed the PRISMA methodology for scoping reviews and systematically searched PsychINFO, PubMed (MEDLINE), ProQuest, Embase and grey literature and reference lists between 1989 and October 2020 for articles related to needle discomfort, distress, anxiety, fear or phobia. The following chronic diseases were included: arthritis, asthma, chronic back pain, cancer, cardiovascular disease, chronic obstructive pulmonary disease, diabetes, and mental illness, or kidney failure. Literature concerning dentistry, vaccination, intravenous drug users and paediatric populations were excluded.

**Results:**

We identified 32 papers reporting prevalence (n = 24), management (n = 5) or both (n = 3). Needle fear prevalence varied in disease cohorts: 17–52% (cancer), 25–47% (chronic kidney disease) and 0.2–80% (diabetes). Assessment methods varied across studies. Management strategies had poor evidence-base, but included needle-specific education, decorated devices, cognitive-behavioural stress management techniques, distraction, and changing the therapy environment or modality.

**Conclusion:**

Although needle fear is common there is a paucity of evidence regarding interventions to address it among adults living with chronic disease. This scoping review has highlighted the need for improved identification of needle fear in adults and development of interventions are required for these cohorts.

## Introduction

People with chronic disease may require frequent and long-term exposure to needles as part of essential disease treatment and, in some instances, to sustain life. People receiving haemodialysis for kidney failure are one such cohort, typically requiring insertion of six large-bore needles/week, or a minimum of 312 needle insertions per year. Chemotherapy or insulin treatments also necessitate multiple injections, infusions, and blood tests over sustained periods.

Needle fear is a common barrier to initiating or adhering to medical treatments [1–3]. Needle fear exists on a continuum of severity from dislike and discomfort to phobia [4]. In the general adult population, the prevalence of injection fear was found to be 16.1% in the Netherlands [5]. The frequency of needle phobia in general adult populations is less common; it was 1.1% in the Netherlands [5], 0.5% in South Korea [6], 1.6% in Sweden [7], and 2.1% in the USA [8]. Both needle fear and needle phobia were found to be more common in women than men [9–11]. Interventions include desensitization therapy [12] and countering vasovagal syncope, for example, by tensing muscles [13]. However, these approaches are not applicable for less severe, yet likely more common needle fear or distress. Exposure-based interventions for the management of individuals with high levels of needle fear across the lifespan have previously been recommended [4]. Needle distress is likely underreported by patients who accept it as the price for staying alive with therapies. It is not systematically measured in clinical care and therefore remains under-recognised and challenging for clinicians and patients to manage.

Existing research concerning needle fear and its management has focussed on paediatric populations [14, 15] or infrequent needle exposure, such as dental procedures [12] and vaccinations [16, 17]. Given the increasing prevalence of chronic disease worldwide [18–20] we synthesised the literature regarding needle fear prevalence and management to assist in clinical care of this cohort.

## Methods

We followed methodology by Arksey and O’Malley [21] and reported according to guidelines for scoping reviews (PRISMA-ScR) [22].

The research question was: ‘What is the prevalence of, and management strategies for, needle fear among adults with chronic disease?’. Search strategy results are defined in Table 1. The search was conducted in October 2020, spanning literature published between January 1 1989 and October 30, 2020, using PsychINFO, PubMed, ProQuest Central, and Embase. Manual searching of reference lists of systematic and other literature reviews identified additional primary studies. Literature was included if it primarily addressed 1) the prevalence of and/or 2) management strategies or recommendations for needle fear in adults (≥18 years) with chronic disease. We defined ‘needle fear’ as needle discomfort, anxiety, fear, distress and/or phobia. ‘Chronic diseases’ were consistent with the World Health Organisation [20] and the Australian Institute of Health and Welfare [23] definitions and included arthritis, asthma, back pain, cancer, cardiovascular disease, chronic obstructive pulmonary disease, diabetes, mental illness and kidney failure. We excluded acute medical conditions, paediatric populations, more infrequent procedures involving needling (e.g. dental procedures, immunisation), intravenous drug use and qualitative studies other than reports discussing management of needle fear.

**Table 1:** Search strategy, terms and results for traditional databases.

Step	Search description	Results			
		EMBASE	PsycINFO	PUBMED	PROQUEST
1	Needle–all fields	186446	11507	134758	376557
2	1 AND anxiety or fear or distress or phobia–title/abstract	2818	910	1775	4553
3	2 AND arthritis or asthma or back pain or cancer or cardiovascular or heart or pulmonary or lung or diabetes or mental illness or psychiatric or renal or kidney or dialysis or renal replacement therapy–title/abstract	968	195	495	1075
**Total**– 2,733					
**Excluding duplicates**– 2342					

Dates included in search: 1 January 1989–30 October 2020. Searches conducted 30 October 2020.

**Fig 1:**
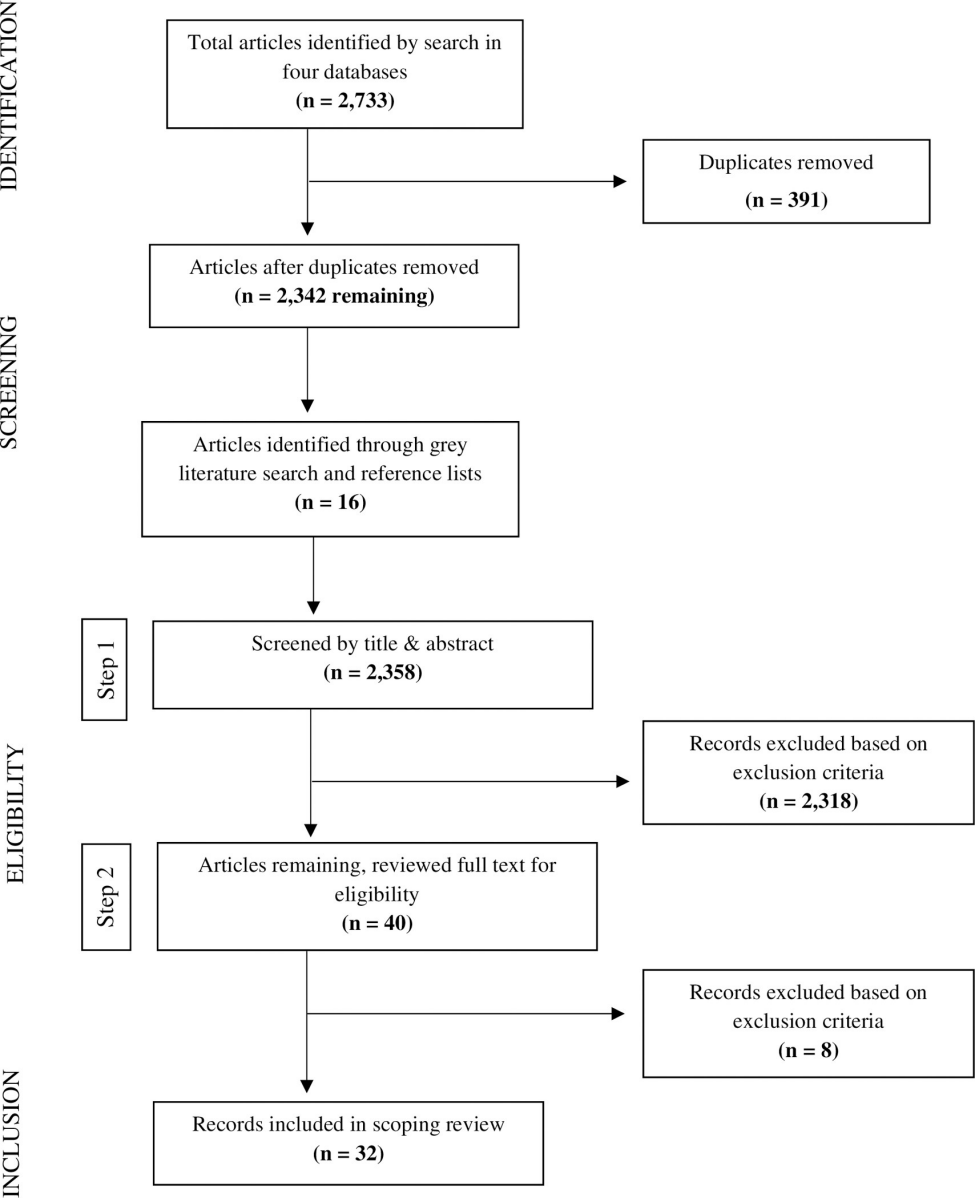
PRISMA flow diagram depicting the article selection process.

The PRISMA flow diagram depicting article selection process is shown in Fig 1. Initial exclusions were made independently by ED and RLL based on title and abstract (Eligibility Step 1). ED reviewed full-text articles for eligibility and conferred with authors RLL and SJ in cases of ambiguity (Eligibility Step 2).

## Results

Thirty-two articles were included (Table 2).

**Table 2:** Findings from included articles reporting needle fear prevalence and/or management strategies and recommendations in adult chronic disease populations.

Author, country		Results		
	Population & Setting	Prevalence of needle fear	Management strategies and recommendations	Instrument/questionnaires used in study
**1a. Cancer (n = 5 articles)**				
Kettwich et al., USA [24]	Paediatric (n = 25) and adult (n = 25) patients receiving chemotherapy across two hospitals in paediatric or adult chemotherapy infusion clinics.	13/25 (52%) of adult patients were overtly needle phobic. Intervention effectively and significantly reduced aversion, anxiety, fear and overall stress, 92% effective (P<0.001) in adults.	Exposure to decorated butterfly needles and syringes (stress-reducing medical devices) used in treatment, compared to conventional devices.	• Visual analogue reaction scales
Cox and Fallowfield, UK [2]	Women with breast cancer (N = 208), including past chemotherapy experience (n = 113), from one of six oncology outpatient clinics	78/208 (38%) reported feeling anxious about injections; 28/208 (13.5%) experienced needle phobia. Among patients with past chemotherapy experience, 52/113 (46%) reported anxiety about injections; 18/113 (16%) had experienced needle phobia.	Changing treatment room, bed or chair, distracting patient away from sight of the cannula, discuss needle anxiety prior to chemotherapy (recommendation only).	• Semi-structured interview • State-Trait Anxiety Inventory (STAI) • Multidimensional Health Locus of Control Scale (MHLOC)
Carey and Harris, Australia [25]	Outpatients receiving intravenous chemotherapy at two major hospitals (N = 197)	36/197 (18%) had ‘strong feelings of fear, disgust or discomfort’ in response to sight of blood or receiving injections, or both, 16/36 (44%) of whom identified in response to injections only.	None	• Fear Survey Schedule (FSS) • Blood Injection Symptom Scale (BISS) • Disgust Scale (DS)
Harris et al., Australia [26]	Outpatients receiving intravenous chemotherapy for the first time (N = 124)	21/124 (17%) experienced blood-injection-injury fear.	None	• Blood Injection Symptom Scale (BISS)
Mackereth et al., UK [27]	Patients receiving intravenous chemotherapy treatments	Not determined.	CALM Framework based on principles of cognitive behavioural therapy: Mindful moist mouth, stress balls and three-step progressive muscle relaxation training. Anxiety-control strategies: Safety behaviours, offering control choices, distraction.	
**1b. Kidney Failure (n = 6 articles)**				
McLaughlin et al., Canada [28]	In-centre haemodialysis patients (N = 173)	Needle phobia 81/173 (47%) most prevalent self-reported barrier to self-care haemodialysis or home haemodialysis.	None	Researcher developed questionnaire • Questionnaire contained 41 items • 1 item related to “needle phobia or no needle phobia”
Mulder et al., Netherlands [29]	In-centre haemodialysis patients at a hospital dialysis department Pilot study (n = 45) Validation study (n = 86)	Pilot: 20/45 patients (44%) reported fear of injection Validation: Participants reported “feeling afraid” 22/86 (26%), “nervous” 36/86 (42%) or worrying 28/86 (33%) the moment the nurse comes to insert the needle. Significantly less needle fear among patients who had been receiving dialysis for a great number of months (p <0.01).	None	• Hospital Anxiety and Depression Scale (HADS) • Diabetes Fear of Injection Questionnaire (DFIQ) • Dialysis Fear of Injection Questionnaire (DFIQ)
Shanahan et al., Australia [30]	Haemodialysis and peritoneal dialysis patients from inpatient and outpatient dialysis units (N = 551)	198/551 dialysis patients (36%) experienced needle fear, 73/208 (37%) of whom indicated needle fear influenced choice of kidney replacement therapy. Needle fear more prevalent among peritoneal dialysis (43%) compared to haemodialysis (35%) patients.	None	Researcher developed questionnaire • Questionnaire contained 2 items: (1) Have you ever been afraid of needles? (2) Has your fear of needles ever impacted on your choice of treatment?
Shafi et al., Pakistan [1]	Patients with Stage 4 CKD and medical indicators of needing haemodialysis, at a nephrology ward of a tertiary care facility (N = 125)	Among 53/125 patients (43%) who refused haemodialysis, “fear of needles” was cited as reason by 13/53 patients (25%).	None	Researcher developed questionnaire • All participating patients were surveyed regarding their prior care, alternative medication use, decision to initiate HD, reasons to accept HD, and reasons to refuse HD. • “Fear of AV fistula needles” was a reason given by patients to refuse HD.
Fernandes, USA [31]	Case report. Haemodialysis patient (Male, 64 years)	Not determined.	Combating phobic response–reclining posture, sustained muscle tension, thinking of anger-provoking situation, biofeedback, imaginal exposure (in-session and homework).	
Fielding et al., UK [32]	Haemodialysis patients	Not determined.	Written and visual information, needle desensitisation, distraction (relaxation), creating a calm environment, visual routines, written arteriovenous access plans.	
Shamasneh et al. [33]	Haemodialysis patients attending outpatient dialysis center (N = 156)	Out of the patients who refused to undergo surgical procedures for dialysis arteriovenous fistula creation (n = 73), 11/73 (15.1%) cited “fear of needles” as the primary reason.	None	• In-person interviews with structured questions.
**1c. Diabetes (n = 20 articles)**				
Feitosa et al., Brazil [34]	Insulin dependent pregnant women with diabetes, attending outpatient endocrine or antenatal clinics (N = 65). Women with pre-gestational diabetes (n = 40)	28/65 (43.1%) were “afraid of needles” at first evaluation.	Multidisciplinary education about diabetes management during pregnancy (not including specific strategies for needle fear). Significant reduction in fear pre- to post-intervention among entire sample. Among women with pre-gestational diabetes: Fear of self-injection reduced from 40% to 15% and Fear of self-testing (blood glucose) from 33% to 15%.	• Diabetes Fear of Injecting and Self testing • Fear of self-injecting insulin (FSI) Questionnaire (D-FISQ) • Fear of Self-Testing (FST)
Berlin et al., France [35]	Type 1 diabetes patients attending outpatient diabetology department (N = 102)	Patients with GHb levels ≥8% scored significantly higher for fear of blood and injury, than those with ≤8%.	None	• General Health Questionnaire • Fear Questionnaire
Celik and Pinar, Turkey [36]	Diabetes patients at ou patient clinic of university hospital (N = 350)	Mean total Diabetes Fear of Injecting and Self-testing Questionnaire (D-FISQ) score was 7.1 ± 2.7 among patients with ‘mild’ anxiety (n = 256), 21.3 ± 7.4 among those with ‘moderate’ anxiety (n = 89) and 32.2 ± 17.1 among those with ‘severe’ anxiety.	None	• Diabetes Fear of Injecting and Self-testing Questionnaire (D-FISQ).
Fu et al., Hong Kong [37]	Type 2 diabetes patients commencing insulin from 15 primary care general outpatient clinics (N = 303)	“Fear of needle injections” most prevalent negative attitude towards starting insulin 205/293 (70%)	None	• Chinese Attitudes to Starting Insulin Questionnaire (Ch-ASIQ)
Heinrich and Callahan, USA [38]	Insulin-treated diabetes patients	Literature search of articles estimates needle fear affects approximately 28% of adult patients managing diabetes.	None	• Literature search
Larkin et al., USA [3]	Insulin naïve diabetes patients (N = 100) from diabetes centre at a general hospital (academic health care facility)	39/100 (39%) of patients reported “fear of self-injection”. 35/100 (35%) feared anticipated “pain with injections”.	None	• Insulin Treatment Appraisal Scale (ITAS)
Makine et al., Netherlands [39]	Insulin naïve diabetes patients (N = 154) from two outpatient clinics at tertiary hospitals	Among patients with low/moderate (n = 113), high (n = 21), and severe (n = 15) depressive symptoms “fear of self-injection” with a needle identified in 43%, 48% and 53%, respectively.	None	• Insulin Treatment Appraisal Scale (ITAS)
Nadasen and Naidoo, South Africa [40]	Diabetes patients (N = 59) from primary healthcare facility	47/59 patients (80%) “afraid of pain” associated with needles. Among insulin refusers despite medical advice 33/59 (56%), all identified as being “afraid of needles and injections”.	None	• Face-to-face interviews with open- and closed-ended questions.
Polonsky et al., USA [41]	Diabetes patients not receiving insulin (N = 708), recruited from a conference for people with diabetes	56/200 (28%) unwilling patients, 50% agreed with “anticipated pain: I couldn’t take the needle every day, it would be just too painful”	None	Researcher developed questionnaire • 9 items examined reluctance to begin insulin therapy • 1 item related to “Anticipated pain I couldn’t take the needle every day; it would be just too painful”
Snoek et al., Netherlands [42]	Insulin-treated diabetes patients (N = 266) recruited from Dutch Diabetic Association	18/266 patients (7%) scored highly on the Fear of Self-Injection and/or the Fear of Self-Testing questionnaire, with 7/18 (39%) of these scoring high on both. Fear of self-testing was significantly lower the longer people had diabetes (*p* < 0.001).	None	• Diabetes Fear of Injecting and Self-testing Questionnaire (D-FISQ)
Zambanini et al., UK [43]	Insulin-treated (≤1 month) diabetes patients (N = 115) attending diabetes outpatient clinic	33/115 patients (28%) had high injection anxiety.	None	• Insulin Treatment Questionnaire
Shafie Pour et al., Iran [44]	Diabetes patients who refused insulin or required administration by professional to continue treatment (N = 214), from a diabetes centre	67/214 patients (31%) reported “fear of needles” as a barrier to insulin initiation.	None	• Insulin noncompliance questionnaire
Mollema et al., 2001a) Netherlands [45]	Insulin-treated diabetes patients (N = 1275) recruited from Dutch Diabetes Association	79/1275 patients (6%) indicated ‘extreme’ scores of fear of self-testing and/or fear of self-injection scales.	None	• Diabetes Fear of Injecting and Self-Testing Questionnaire (D-FISQ)
(Mollema et al., 2001b) Netherlands [46]	Insulin-treated diabetes patients (N = 24), recruited from Dutch Diabetes Association	0.2–1% of population scored in the severe range for “fear of injection”. 0.6.-0.8% of total study population scored above cut-off score for “fear of self-testing”.	None	• Diabetes Fear of Injecting and Self-Testing Questionnaire (D-FISQ)
Mollem et al., 1996) Netherlands [47]	Insulin-treated diabetes patients (N = 240) of outpatient hospital clinic	6/240 patients (3%) had scores of ≥ 3 for being “afraid of injecting” self (indicating serious problems).	None	• Barriers in Diabetes Questionnaire (BDQ)
Zambanini and Feher, UK [48]	Case report. Diabetes patient with long-standing needle phobia (Female, 33 years), attending outpatient clinic	Not determined.	Behavioural modification techniques, topical anaesthetic cream, rubbing the area, providing reassurance and education about anxiety, pre-medication, offer alternative therapies	
Brunton et al., USA [49]	Type 2 diabetes patients	Not determined.	Discuss patient fear when educating about insulin therapy, provide reassurance of physical characteristics of devices, desensitization techniques by behavioural counsellor, and discuss alternative treatment options.	
Chen et al., China [50]	Type 2 diabetes patients receiving injectable antidiabetic therapies (IAT) or had received IAT continuously for at least 1 month (N = 500)	185/500 patients (37%) reported “fear of injection”.	None	Researcher developed questionnaire • 31 questions in the form of single-choice questions, multiple-choice questions, and text-entry questions • The patient survey assessed concerns of initiating IAT, satisfaction with IAT, aspects of IAT that need improvement, and IAT training received. • Under patient concern, 1 item included “fear of injection”
Sharma et al., India [51]	Type 2 diabetes patients who visited diabetic clinic and were prescribed insulin for the first time in last 2-year period (N = 225)	Of the 105/225 patients (47%) who delayed insulin treatment 37/105 (35%) reported “needle phobia” due to fear of pain.	None	• Barriers to Insulin Treatment (BIT) Questionnaire
Ngassa Piotie et al., South Africa [52]	Type 2 diabetes patients (N = 468)	348/468 patients (75%) reported being “afraid of needles”. As a barrier to insulin therapy 310/468 patients (67%) were “scared of needles and the pain from injections”.	None	Researcher developed questionnaire • Psychological insulin resistance was assessed using 19 items • 1 item included “I am afraid of needles”

### Article characteristics

Articles included 24 cross-sectional studies [1–3, 25, 26, 28–30, 33, 36, 37, 39–45, 47, 50–52], one abstract of a literature review [38], one prospective cohort study [34], two case reports [31, 48], one randomized controlled trial [24], and three reports [27, 32, 49]. The included studies used a variety of methodology to define and assess needle fear or phobia, ranging from a single question within a broader questionnaire, face-to face interviews to assessment of fear or phobia using a dedicated validated tool (eg. Diabetes Fear of Injecting and Self-testing Questionnaire D-FISQ or the Blood-Injection Symptom Scale (BISS)). This made interpretation between studies difficult.

Twenty-four articles assessed prevalence of needle fear [1–3, 24–26, 28–30, 33–36, 38–45, 47, 50–53], five described only management strategies or recommendations [27, 31, 32, 48, 49] and three reported both prevalence and management [2, 24, 34]. Of the eight articles that suggested management recommendations, three were underpinned by research evidence [27] or outcome data from quasi-experimental study [34] and randomised-controlled trial [24].

Twenty articles addressed diabetes [3, 34–44, 46–53], seven addressed chronic kidney disease [1, 28–33], and five addressed cancer [2, 24–27].

### Settings and populations

The majority of studies originated from USA, Netherlands and the UK in outpatient settings.

### Prevalence of needle fear

#### Cancer

Prevalence of needle fear ranged from 17–52% [2, 24–26] among adults with past or current experience of chemotherapy (Table 2, section 1a). Two studies explored feelings of disgust, fear or discomfort at the sight of blood or receiving injections, with needle fear experienced by 21/124 (17%) [26] and 36/197 (18%) [25]. Self-report measures of ‘needle fear’ included the Blood-Injection Symptom Scale (BISS) [26], the Blood-Injection Injury Scale [25], and a semi-structured interview [2]. ‘Needle phobia’ was determined by a score of ≥5 across visual analogue scales of anxiety, fear, aversion, and stress [24].

#### Kidney failure

Needle fear was reported by 25–47% of adults receiving peritoneal dialysis or haemodialysis (Table 2, section 1b) [1, 28–30]. Mulder et al.’s [29] validation study of the Dialysis Fear of Injection Questionnaire (DFIQ) in haemodialysis patients revealed 20/45 (44%) had fear of needles in the pilot study. In the validation component (n = 86), participants reported feeling afraid 22/86 (26%), nervous 36/86 (41%) or worried 28/86 (33%) the “moment the nurse comes to insert the needle”.

In response to two researcher-administered questions, 198/551 (36%) of peritoneal and haemodialysis patients reported experiencing needle fear, 73/208 (37%) of whom indicated this had influenced their choice of kidney replacement therapy [30]. 13/53 (25%) of people who refused haemodialysis cited fear of needles and complications as a reason [1]. Needle phobia was the most prevalent barrier to self-care haemodialysis with 81/173 (47%) reporting needle phobia from a researcher developed questionnaire [28]. Fear of needles was cited as a barrier toward arteriovenous fistula creation and use in 11/73 (15%) of haemodialysis patients that underwent in-person interviews [33].

#### Diabetes

Across four primary studies of adults with insulin-treated diabetes, the Diabetes Fear of Injecting and Self-testing Questionnaire (D-FISQ) yielded estimates of needle fear between 0.2–43% (Table 2, section 1c) [34, 42, 45, 46]. In 350 diabetes patients, D-FISQ scores were greater in patients with severe levels of anxiety, assessed by the State Anxiety [36]. A literature review abstract which searched articles related to prevalence of needle fear, needle phobia, injection fear or blood-injury-injection phobia suggested needle fear affects 28% of patients receiving insulin injections, however no details of assessment methods were included [38].

Among patients receiving insulin, 6/240 (3%) scored ≥ 3 on items “I am afraid of injecting myself” and “I’m afraid to prick my finger” using the Barriers to Diabetes Questionnaire, indicating “serious problems” with needles [47]. A study utilising face-face interviews examined the prevalence of primary non-adherence with insulin and barriers to insulin initiation in patients with type 2 diabetes [51]. This study revealed that 47% (105/225) delayed insulin treatment. Of the 105 patients who delayed treatment, 35% (37/105) reported needle phobia due to fear of pain. In a study of Type 2 diabetes patients receiving injectable antidiabetic therapies (IAT), 185/500 (37%) of patients were afraid of injection and felt fear when thinking of a needle [50]. In the same study, 137/200 (67%) Endocrinologists reported via questionnaires that fear of injection was a major concern for patients for initiating injectable treatment [50].

Interviews with patients with Type 2 diabetes revealed 47/59 (80%) were afraid of pain associated with needles and injections; this was 100% in a sub-sample (n = 32) who refused insulin therapy despite medical advice [40]. A “fear of needle injections”, an item on the Chinese Attitudes to Starting Insulin Questionnaire, was the most prevalent negative attitude towards starting insulin in 205/293 (70%) of patients with type 1 diabetes [37]. Patients treated with insulin 33/115 (28%) for less than one month had a high injection anxiety score on an author-created questionnaire, 16/115 (14%) of whom had avoided injections and 48/115 (42%) indicated they would be “troubled by more frequent injections” [43].

Fear of self-injection or anticipated pain with injections was reported by 35–53% of diabetics without experience of insulin [3, 39]. Of non-insulin dependent patients with type 2 diabetes 56/200 (28%) were unwilling to receive insulin, with 50% citing anticipated pain and “inability to take” needling every day as a key reason (although not clear if this was due to distress or anxiety) [41]. Similarly, 67/214 (31%) diabetic patients who initially refused insulin or who received insulin administration by a physician indicated fear of needles as a barrier [44]. Among Type 2 diabetic patients more than half 243/468 (52%) expressed unwillingness to start insulin therapy, the attitudinal items from the administered questionnaire that most strongly distinguished unwilling from willing participants included injection-related anxieties including fear of needles, with 218/243 (90%) of unwilling patients being afraid of needles compared to 130/225 (58%) of willing patients [52]. In 102 patients with type 1 diabetes, those with high scores for fear of blood and injury as measured by The Fear Questionnaire [54], performed fewer blood glucose measurements and had poorer glycaemic control than patients without fear [35].

### Management strategies and recommendations

Eight sources described strategies or recommendations for needle fear management: one RCT of 25 adult chemotherapy patients [24], one report of a treatment protocol [27], one cross-sectional survey of 208 women with breast cancer [2], two case reports [31, 48], one recommendation report [32], one narrative report [49], and one prospective cohort study of 65 pregnant women predominantly with pre-gestational diabetes [34].

#### Cancer

Kettwich et al. [24] delivered an intervention among 25 adults receiving chemotherapy including random exposure to conventional or stress-reducing needles and syringes (decorated barrel with colourful glitter stickers) (Table 2, Section 1a). Emotional responses were measured using visual analogue scales of anxiety, aversion, fear and overall stress (score range 0–10, higher scores indicating greater fear [24]. Fifty-two percent of the sample had a phobia of butterfly needles pre-intervention (determined by a score of 5 or more). Among this group, a 92% reduction (P<0.001) of aversion, anxiety, fear and overall stress was observed when exposed to stress-reducing compared to conventional devices, however no follow-up data was collected.

The ‘CALM’ treatment guide study included rapid stress management techniques for patients receiving intravenous chemotherapy, including mindful moist mouth, stress balls, and progressive muscle relaxation [27]. Other “anxiety control strategies” included safety behaviours (e.g. medication, presence of caregiver), offering control choices (e.g. taking a break during procedures) and distraction. Authors noted suggestive language, namely “discomfort warnings” from health professionals contributed to patient discomfort. No evaluation of strategies was described.

Cox & Fallowfield [2] recommended nursing staff “vary” the treatment environment (e.g., room, bed, chair), distract the patient away from the sight of needles and provide opportunity to discuss needle anxiety prior to chemotherapy; however implementation and evaluation was not conducted.

#### Kidney failure

Rapid exposure and desensitisation was applied over three sessions to reduce needle phobia in a 64-year-old male requiring dialysis, resulting in reduced subjective distress [31]. Strategies included reclining posture, sustaining tension of facial muscles and extremities and thinking of a situation triggering anger. Biofeedback through blood pressure and pulse monitoring demonstrated changes in blood pressure and pulse. Imaginal exposure implemented by a therapist included simulation of needling. Twice-daily exposure tasks were self-directed by the patient at other times.

The British Renal Society made recommendations for management of patient needle anxiety based on consensus opinion of 15 nurses from 13 UK dialysis units and research evidence where available [32]. Recommendations included written information, photographs and illustrations to prepare patients for needling, desensitisation, written arteriovenous access plans, visual routines, distraction through relaxation, and creating a calm environment. Authors highlighted the importance of listening, trust between patients and staff, and use of coping strategies, although these were not described.

#### Diabetes

Feitosa et al. [34] evaluated a multidisciplinary diabetes education program (nurses, endocrinologists, dieticians and obstetricians) on fear of self-injecting and testing among 65 women with pre-gestational or gestational diabetes taking insulin during pregnancy. Education included the impact of hyperglycaemia, diet and lifestyle, and training on self-monitoring of blood glucose and insulin injection administration. Women completed the short D-FISQ at the first review and within the last two weeks of pregnancy or postpartum. Needle fear was identified in 43.1% of participants’ pre-intervention. Post-intervention, fear of self-injection significantly reduced from 39% to 13% (*p* = 0.001) and similarly, fear of self-testing from 28% to 14% (*p* = 0.012), despite no specific strategies for needle fear in the intervention. Among women with pre-gestational diabetes specifically, fear of self-injection reduced from 40% to 15% and fear of self-testing (blood glucose) from 33% to 15%, (not statistically significant).

Strategies to address needle fear as a barrier to insulin use included describing physical characteristics of needles and insulin pens to patients and in cases of “severe fear” (undefined), informing patients of alternative insulin pumps or desensitization techniques by a “behaviour counsellor” (undefined) [49]. For a 33-year-old woman with Type 1 diabetes, management strategies included behavioural modification techniques, topical anaesthetic cream, education about anxiety, pre-medication and offering alternative therapies (jet injection devices without needles) [48].

## Discussion

This review has demonstrated the high prevalence of needle fear and distress among adults with cancer, diabetes or kidney failure. We identified 32 diverse heterogeneous articles with variable scientific methodology, ranging from non-evidence-based recommendations to one randomised controlled trial. Only eight studies addressed the management of needle fear, indicating it is under-researched in adult chronic disease populations. Even fewer of these provided sufficient detail about strategies, including timing and frequency of delivery, or formal evaluation. This dearth of evidence may reflect a lack of recognition of the seriousness of this issue for patient well-being, where refusal or avoidance of treatments may result in reduced quality of life, reduced lifespan, or death. Whilst needle fear is frequently cited as a reason for treatment avoidance among such groups [1–3], the review highlights the need for high-quality evidence of strategies or interventions to enable better management of this problem in clinical care.

The frequency of needle fear varies widely across studies–from 0.2–80% among patients with diabetes, 17–52% in those with cancer and 25–47% in those with kidney failure. Variability in the prevalence of needle fear may due to differences in the underlying demographics, frequency of medical procedures or characteristics of the patients [9, 24]. The diversity of assessment measures used to identify needle fear may have contributed to the variation in frequency between studies. The D-FISQ and its variant, the DFIQ, were used among patients with diabetes or kidney failure, as a validated measure of fear of injection and or self-testing associated with insulin or dialysis therapies [29, 34, 36, 42, 45, 46, 53]. However, a variety of other assessment methods were used including psychometric self-report tools [25, 26, 35, 39], investigator-created surveys [1, 28, 30, 41, 50, 52], and face-to-face interviews [2, 33, 40]. Moreover, needle phobia was determined by single or multiple items on self-report measures [2, 24, 28, 51], not formal diagnostic assessment.

The prevalence estimates yielded suggest a need for routine screening of needle fear via validated patient-reported measures in chronic disease cohorts where needle exposure is high. Measures that assess needle fear in the context of a therapy, such as the D-FISQ, should be utilised where available. Screening may be warranted particularly prior to treatment initiation, as needle fear is associated with treatment refusal [1, 33, 40, 44, 52], or in the early weeks and months of therapy, when fear appears heightened [1, 42, 43, 55]. Early identification of patient fear or misconceptions of therapy could aid in treatment-decision making and provide opportunity for education and early intervention. This would likely reduce patient distress, improve treatment adherence and prevent resulting complications. Where standardised measures are not available or feasible, simple questions about the patient’s preferences for treatment, including regarding needles, validates their concerns and allows opportunity for discussion about how fears may be managed.

Aversion to needles can be conceptualised on a continuum, from fear, to more serious presentations warranting the clinical diagnosis of phobia [56]. In this review the distinction between needle fear, fear of pain from needles and a diagnosed anxiety disorder such as phobia, was seldom made by authors; rather these terms were used interchangeably. Consistent with the continuum of distress, management may range from simple, targeted interventions to an intensive psychological treatment program. Individuals with a formally diagnosed needle phobia will require the latter; delivered by appropriately trained clinicians, such as psychologists or psychiatrists. Evidence exists from controlled trials for the effectiveness of exposure therapy and desensitization therapy for such individuals [4, 15, 32, 57] and was recommended by some articles reviewed here [31, 32] however there was a lack of detail of therapeutic activities, nor was there evidence of evaluation of their application in the context of different chronic disease therapies from the selected peer review studies in this scoping review.

For individuals with mild to moderate fear of needles, potential psychological interventions may include education or cognitive behavioural therapy programs inclusive of relaxation and cognitive restructuring techniques. In the current review, psychological strategies to reduce fear included stress management, distraction, and relaxation [2, 27, 32]. Education about treatment and needling, including use of visual and written materials was recommended [32, 34, 48], however only one study described formal evaluation of an educational program which resulted in a reduction in needle distress, despite not including targeted interventions for this [34]. Therapeutic modifications, such as changes to needling devices and offering alternative treatments (i.e. without needles) were also recommended [2, 24, 49]. Despite these management strategies being identified in the current review, there is a lack of high-quality evidence of intervention protocols to prevent or alleviate needle fear among adults with chronic disease, with very little to no replication of findings. This may be in part due to under recognition of patient needle distress among care providers and lack of systematic screening. There was often overlap of fear of pain and fear of needles within selected.

Further longitudinal assessment of needle fear over the course of disease and treatment is needed, as are randomised trials of interventions to address this, tailored to the specific context and features of chronic disease therapies.

This review did not find research articles relevant to the fear of needles in patients with arthritis, asthma, chronic back pain, cardiovascular disease, chronic obstructive pulmonary disease, or mental illness. Such patients also require clinical evaluation through laboratory testing which may require periodic blood draws. Moreover, hospitalization may be required which involves invasive procedures. It is important that future research on needle fear be conducted in these patients as well.

This review has some limitations. The search strategy utilised Australian Institute of Health and Welfare [23] terms for the chosen chronic diseases of interest, and wild cards were not used. We only included studies published in English between 1989 and 2020.Due to heterogeneity of articles identified we were unable to grade the evidence or make definitive conclusions regarding appropriate assessment tools for needle fear or strategies for clinical care.

This review has highlighted that needle fear is a significant problem for adults managing chronic disease, particularly cancer, kidney failure and diabetes. It has the ability to erode long-term health by undermining the initiation of, or engagement in, life-sustaining treatments and contributes to the psychological burden associated with chronic disease management. Better understanding of the factors associated with the origins and promoters of needle fear is needed. The development and evaluation of effective treatments is urgently required in order to improve the physical and psychological wellbeing of adults living with chronic disease with frequent needle exposure.
